# Leading Teachers’ Emotions Like Parents: Relationships Between Paternalistic Leadership, Emotional Labor and Teacher Commitment in China

**DOI:** 10.3389/fpsyg.2020.00519

**Published:** 2020-04-03

**Authors:** Xin Zheng, Xiao Shi, Yuan Liu

**Affiliations:** ^1^Center for Studies of Education and Psychology of Ethnic Minorities in Southwest China, Southwest University, Chongqing, China; ^2^Faculty of Education, Southwest University, Chongqing, China; ^3^Faculty of Psychology, Southwest University, Chongqing, China

**Keywords:** emotional labor, paternalistic leadership, teacher commitment, Chinese contexts, mediation analysis

## Abstract

Emotional labor plays an essential role in school leadership and teaching, as principals and teachers undergo complex interactions with students, colleagues, and parents. Although researchers have realized the influence of leaders’ behaviors on followers’ emotions in management and educational contexts, the relationship between leadership behaviors, teachers’ emotional labor, and related organizational outcomes has been underexplored. As leadership and emotional labor are situated and influenced by cultural contexts, the current study focused on the relationship between teachers’ emotional labor strategies, multidimensional teacher commitment, and paternalistic leadership, a unique leadership type rooted in Confucianism. Paternalistic leadership is a style that combines strong authority with fatherly benevolence, which is prevalent in East Asia and the Middle East. A sample of 419 teachers was randomly selected to participate in a survey. The results showed that principals’ authoritarian leadership behaviors had negative influences on teachers’ commitment to the profession and commitment to the school. Benevolent leadership had positive effects on teachers’ commitment to students, commitment to the profession, and commitment to the school. Teachers’ deep acting played positive mediating effects, while surface acting was a negative mediator. The results imply that school leaders could properly exert parent-like leadership practices to facilitate teacher commitment through managing teachers’ emotions.

## Introduction

Emotional labor plays an essential role in school leadership and teaching, as principals and teachers undergo complex interactions with students, colleagues, and parents. According to Hochschild (1983), emotional labor refers to “the management of feeling to create a publicly observable facial and bodily display” (p. 7). There are two widely recognized emotional labor strategies in emotional labor research: surface and deep acting (Hochschild, 1983; Grandey, 2000). Surface acting refers to the strategy by which employees modify their emotional expression to comply with organizational rules, while deep acting means the process of changing one’s internal feelings to display the required emotional expression by using some cognitive techniques (Brotheridge and Lee, 2003; Grandey, 2003). Thus, interpersonal relationships and interactions are essential for understanding emotional labor. Most current studies of emotional labor have focused on the relationship between employees and the clients, but a paucity of studies has explored the emotional labor caused by interactions with leaders (Hülsheger and Schewe, 2011; Grandey and Melloy, 2017). For example, in educational contexts, research on teachers’ emotional labor mainly has focused on their interactions with students (Uitto et al., 2015; Berkovich and Eyal, 2017). As employees perform emotional labor to meet organizational rules specific to their roles (Hochschild, 1983; Brotheridge and Lee, 2003), teachers may perform different emotional labor strategies when they interact with students and principals. Studies have shown that leaders play a key role in influencing subordinates’ emotional labor (Humphrey et al., 2008, 2015; Gooty et al., 2010). Different leadership behaviors may evoke followers’ emotional display in various organizations (Bozionelos and Kiamou, 2008; Humphrey et al., 2016). However, a lack of studies in management (Grandey and Melloy, 2017) and educational field (Uitto et al., 2015; Berkovich and Eyal, 2017) has explored the effect of leadership practices on followers’ emotional labor. As Grandey and Melloy’s (2017, p. 417) recent review pointed out, “surprisingly little research has explored specific managerial practices and their effects on emotional labor and outcomes.”

In the field of school leadership, studies in the past four decades have elicited some agreement. First, there are no “fit for all” leadership models and “successful leaders are sensitive to the contexts in which they enact different leadership practices as contexts change” (Leithwood et al., 2019, p. 5). Second, school leaders influence teaching and student learning indirectly through improving staff motivation, ability, emotions, and working conditions (Hallinger, 2011; Leithwood et al., 2019). For example, Leithwood et al. (2017) summarized four paths by which school leadership influenced student achievement and teacher learning: rational, emotional, organizational, and family paths. Third, researchers have argued that successful leadership practices can hardly escape from the cultural context (Hallinger, 2011; Leithwood et al., 2017; Walker and Qian, 2018). Studies of school leadership have been dominated by instructional leadership and transformational leadership, both of which originated in Anglo-American contexts. Since the 2000s, calls were made to move from the Anglo-American axis of influence and develop more international and contextually bounded scholarship characteristics by a multiplicity of voices (Dimmock and Walker, 2000). In the growing literature on leadership in non-Western countries, one form of leadership style that is prevalent but often ignored is paternalistic leadership (PL; Jackson, 2016; Bedi, 2019).

Paternalistic leadership is a leadership style that combines leader authoritarianism and benevolence (Farh and Cheng, 2000; Aycan, 2006; Cheng et al., 2014). Authoritarian leadership (AL) refers to behaviors that assert absolute authority and control over the subordinates and demand unquestionable obedience from them. Authoritarian leaders also set an expectation of high standards and punish employees for poor performance (Tian and Sanchez, 2017; Wang and Guan, 2018). Benevolent leadership (BL) refers to behaviors that show individualized, holistic concern for subordinates’ professional, personal, and familial well-being (Farh and Cheng, 2000). PL is rooted in the Confucianism culture, which emphasizes hierarchical status and interpersonal relationships. Although the “control” and “care” roles seem to be paradoxical/controversial, they can be present at the same time, similar to the way a father treats his child (Farh and Cheng, 2000; Aycan, 2006). As Aycan (2006) argued, paternalism is most likely to occur in cultures characterized by collectivism (vs. individualism), high power distance (vs. low), and high affectivity (vs. emotional neutrality). Even though the concept of PL was originally described in Chinese firms, scholars have noted or examined its existence not only in East Asia but also in Latin America and the Middle East, for example, Mexico and Turkey (Pellegrini and Scandura, 2008; Hiller et al., 2019).

Given that instructional leadership is generally task oriented, which primarily focuses on curriculum and instruction to improve student outcomes (Hallinger, 2011), PL is mainly relationship oriented and culture specific (Farh and Cheng, 2000; Pellegrini and Scandura, 2008; Chen et al., 2014). As the relationship between a paternalistic superior and subordinates is “a heavily emotional one” (Aycan, 2006, p. 452), paternalistic leaders can induce various emotional reactions from followers. For example, emotions induced by PL are often related to respect, liking, gratitude, or fear (Farh et al., 2006; Chen et al., 2014). Previous studies have shown that general leadership practices (e.g., developing people, restructuring organization, and setting direction) influenced teachers’ emotional labor strategies (Zheng et al., 2018). Only a few studies have directly explored the relationship between PL and subordinates’ emotions or emotional response. For example, Karakitapoğlu-Aygün et al. (2019) found that BL influenced follower performance through evoking positive emotions. Wu et al. (2002) found that AL evoked angry emotions (i.e., anger, indignation, and agitation) and tended to suppress the expression of such negative emotions. To date, it is not known how such a culturally specific leadership style influenced teachers’ emotional labor strategies, as PL is prevalent in Chinese schools (Farh et al., 2008).

Following the suggestion that future research should explore the relationship between emotional labor strategies, leadership styles and followers’ well-being, work attitudes, and job performance in various contexts (Hülsheger and Schewe, 2011; Berkovich and Eyal, 2015), this study explored the effects of PL on teachers’ emotional labor and organizational outcomes. In terms of organizational outcomes, this study selected teacher commitment, which is frequently reported as an important indicator for school effectiveness (Firestone and Pennell, 1993; Park, 2005; Meyer et al., 2012). Educational policymakers and researchers concern teacher commitment frequently as it is highly correlated with teacher turnover rate (Firestone and Pennell, 1993; Park, 2005). The study defines commitment as the psychological bond or identification of the individual with an object (Somech and Bogler, 2002; Park, 2005). The objects of commitment could vary (Park, 2005; Chan et al., 2008). There are three major objects of teacher commitment: commitment to their school, commitment to the teaching profession, and commitment to students. Teacher commitment to school and teacher organizational commitment are often interchangeably used, which is defined as the relative strength of the identification of the individual and his or her involvement in a particular organization (Mowday et al., 1979). Teachers who are committed to a school have strong beliefs in the school’s goals and values, and tend to remain in the school (Chan et al., 2008; Meyer et al., 2019). Teacher commitment to the teaching profession is a positive affective attachment to one’s occupation (Somech and Bogler, 2002; Park, 2005). This indicates the extent of a person’s identification and satisfaction as a teacher (Park. 2005; Somech and Bogler, 2002; Razak et al., 2010). Teacher commitment to students is defined as teacher devotion to and responsibility for student learning and behavior (Park, 2005; Lee et al., 2011). These three dimensions are different from each other. For example, even if teachers are not committed to the organization, they can still be committed to their work and their students (Frelin and Fransson, 2017). A teacher who is highly committed to teaching profession may have low commitment to the school when he/she is unsatisfied with the principal or the school goals (Somech and Bogler, 2002; Meyer et al., 2019). The study used such a multidimensional construct of teacher commitment.

Some current studies documented the relationship between PL, emotional labor, and commitment. Commitment, loyalty, and decreased turnover are frequently reported benefits of paternalism for employers (Farh et al., 2006; Chen et al., 2014; Zhang et al., 2015). Generally, benevolent behaviors have been found to be positively related to commitment to the team, affective and continuance commitment, deference to supervisor, and job satisfaction (Cheng et al., 2002; Erben and Guneser, 2008; Chen et al., 2014; Bedi, 2019). For AL, the general consensus in the literature is that authoritarian tendencies are associated with negative behaviors and outcomes (Pellegrini and Scandura, 2008; Bedi, 2019). For example, Farh et al. (2008) found that AL has a negative effect on employee organizational commitment. Therefore, we hypothesized that AL (Hypothesis 1a) and BL (Hypothesis 1b) would be significantly related with teacher commitment.

A few studies documented the relationship between commitment and emotional labor. Teachers’ commitment is perceived to have an emotional base (Berkovich and Eyal, 2017). According to Meyer et al. (2019), commitment can reflect an emotional attachment (affective commitment) to specific targets. Emotional labor reflects one’s emotional management, displaying emotions in response to organizational rules and interactions with actors (Hochschild, 1983; Brotheridge and Lee, 2003). Strict emotional display rules on one’s job may reflect on the attitudes of the job inclement toward the organization, influencing one’s commitment toward it (Bozionelos and Kiamou, 2008; Huang et al., 2019). Thus, the study considered teachers’ commitment as an outcome of their emotional work (including emotional labor) in school. Studies in management contexts showed that surface acting might negatively affect task performance, by impairing job attitudes such as organizational commitment (Judge et al., 2001; Riketta, 2002). Surface acting displayed substantial negative relationships with organizational attachment, while deep acting had a positive relationship with organizational attachment (Hülsheger and Schewe, 2011). Bozionelos and Kiamou (2008) found that surface acting was a significant predictor for organizational commitment. Ghalandari et al.’s (2012) study of the hospital sector found that deep acting positively influenced organizational commitment while surface acting was not a significant predictor for organizational commitment. The studies cited above were mostly conducted in business or hospital contexts. The current study attempted to explore the relationship between PL, teachers’ emotional labor, and teacher commitment in school contexts. Thus, the second alternative hypothesis was proposed that surface acting (Hypothesis 2a) and deep acting (Hypothesis 2b) would be significantly associated with teacher commitment.

In addition, the study followed the argument that school leaders influenced teaching and learning through multiple paths, one of which is the emotional path (Hallinger, 2011; Leithwood et al., 2017). According to this theory, principals’ behaviors may influence teachers’ work attitudes such as commitment, indirectly through their emotional interactions with teachers. McColl-Kennedy and Anderson (2002) suggested that followers’ emotional regulation or appraisal of emotion might mediate the relationship between leaders’ behavior and followers’ performance. Frijda (2008) also argued that appraisal of emotion functions as a mediating process, compelling the individual toward a particular behavior. These arguments led some researchers to examine the mediating effects of emotion-related variables on the relationship between leadership practices and organizational outcomes. For example, in school contexts, Berkovich and Eyal (2017) found that the effects of transformational leadership on teachers’ organizational commitment were partially mediated by emotional reframing. Zheng et al. (2018) found that the effects of leadership practices on teacher self-efficacy were significantly mediated by surface acting and deep acting. In management studies, Ashforth and Kreiner (2002) argued that emotional reframing or emotional labor strategies in manager–employee interactions may enhance followers’ sense of integration to the organization. In a most recent meta-analysis of PL, Bedi (2019) suggested that future research should further explore the mediating role of some psychological mechanisms in the relationships between PL and employee outcomes. Thus, the study explored the mediating role of emotional labor strategies on the effects of leaders’ paternalistic behaviors and teacher’s multidimensional commitment. The third hypothesis was proposed: emotional labor strategies significantly mediated the effects of PL and teacher commitment (Hypothesis 3).

## Methods

The current study aims to explore the relationship between PL, emotional labor, and teacher commitment in a Chinese school context, with a particular focus on the mediating role of emotional labor strategies. Quantitative methods were used to test the hypothesized relationships mentioned above. A total of 419 teachers from elementary schools in southern China were investigated from October 2018 to March 2019. The teachers were randomly selected when they joined the professional training programs in teacher colleges or universities. Using a convenient sample, the researchers asked voluntary teachers to complete a questionnaire. Before the participants filled the questionnaire, they completed a written informed consent form, which is approved by the first author’s University Survey Research Ethics Committee. The questionnaire was administered by the authors. The participants consisted of 89 males (21.2%), 325 females (77.4%), and 5 missing values; 180 (42.9%) of the teachers taught the Chinese language, 126 (30.0%) teachers taught mathematics, 104 (26.9%) teachers taught other subject (English, science, music), and 9 teachers did not report their subject. In terms of their teaching experience, 102 (24.3%) had taught for 7 years or less, 96 (22.9%) had taught for 8–15 years, 104 (24.8%) had taught for 16–23 years, and 90 (21.4%) had taught for 24 years or more; 98 (23.3%) teachers are from rural schools, while 319 (76.5%) teachers are from urban or suburban schools.

A questionnaire consisting of three scales, namely, the Paternalistic Leadership Scale (PLS), the Teacher Emotional Labor Strategy Scale (TELSS), and the Teacher Commitment Scale (TCS), was used in this study. The PLS was adapted from Cheng et al. (2014) and contained two subscales: Authoritarian Leadership (AL, five items) and Benevolent Leadership (BL, five items). The teachers rated each item on a six-point Likert scale ranging from “strongly disagree” to “strongly agree.” The TELSS was validated by Yin et al. (2017) in a Chinese context. Surface acting includes six items and deep acting includes four items. Teachers rated each item on a five-point Likert scale ranging from “strongly agree” to “strongly disagree.” The 17-item TCS was adapted from Razak et al. (2010). The scale has three dimensions: teacher commitment to school (CSC, five items), teacher commitment to students (CST, five items), and teacher commitment to the profession (CP). Participants rated each item on a five-point scale from “strongly disagree” to “strongly agree.” PLS and TELSS were developed in Chinese and TCS was in English. All three scales have been used and validated in Chinese contexts (Cheng et al., 2014; Han et al., 2016; Zheng et al., 2018).

We used SPSS 19.0 and Mplus 7.0 to analyze the data. First, a confirmatory factor analysis (CFA) was conducted to examine the construct validity for each scale. We then calculated the descriptive statistics (*M* and *SD*) and correlations using SPSS. The hypothesis was tested through the structural equation modeling (SEM) method and mediation analysis using Mplus. The indices that indicate the robustness of fit include the chi-square statistic (χ^2^), the root mean square error of approximation (RMSEA), the Tucker–Lewis index (TLI), and the comparative fit index (CFI). In terms of the criteria of an acceptable data fit, a combination of CFI >0.90, TLI >0.90, and RMSEA <0.1 was used as the cutoff (Hu and Bentler, 1999). Further, a bootstrapping method was conducted to detect mediation effects (Hayes, 2009).

## Results

### Reliability and Construct Validity of the Scales

We first examined the reliability and construct validity of the scales. All seven factors had acceptable reliability coefficients, and their Cronbach’s alpha coefficients ranged from 0.68 to 0.89 (see Table 1). For the PLS, the two-factor structure of PL showed a good data fit (χ^2^ = 345.63, *df* = 64, *p* < 0.01, RMSEA = 0.100, CFI = 0.98, TLI = 0.97), with factor loadings ranging from 0.57 to 0.93. TELSS also showed a good data fit (χ^2^ = 212.12, *df* = 34, *p* < 0.01, RMSEA = 0.110, CFI = 0.97, TLI = 0.96). For the TCS, the results showed an excellent data fit (χ^2^ = 190.67, *df* = 74, *p* < 0.001, RMSEA = 0.062, CFI = 0.99, TLI = 0.99). The descriptive statistics and the correlation results of the seven factors are displayed in Table 1. As shown, the correlations among them were all significant. Therefore, hypothesis 1 and hypothesis 2 were supported.

**TABLE 1:** Descriptive statistics, Cronbach’s α, and correlation matrix.

	1	2	3	4	5	6	7
1. AL	−	−	−	−	−	−	−
2. BL	−0.35***	−	−	−	−	−	−
3. DA	−0.15**	0.39***	−	−	−	−	−
4. SA	0.38***	−0.26***	0.11*	−	−	−	−
5. CSC	−0.38***	0.65***	0.38***	−0.24***	−	−	−
6. CST	−0.30***	0.57***	0.38***	−0.25***	0.78***	−	−
7. CP	−0.35***	0.53***	0.31***	−0.16***	0.66***	0.62***	−
*M*	2.75	5.32	4.13	2.33	4.76	4.83	4.63
*SD*	1.23	0.93	0.82	1.08	0.45	0.37	0.57
Cronbach’s *alpha*	0.75	0.80	0.70	0.85	0.86	0.87	0.76

*p < 0.05, **p < 0.01, ***p < 0.001. AL, authoritarian leadership; BL, benevolent leadership; DA, deep acting; SA, surface acting; CSC, commitment to school; CST, commitment to students; CP, commitment to the profession.

### Structural Equation Modeling Results

**FIGURE 1:**
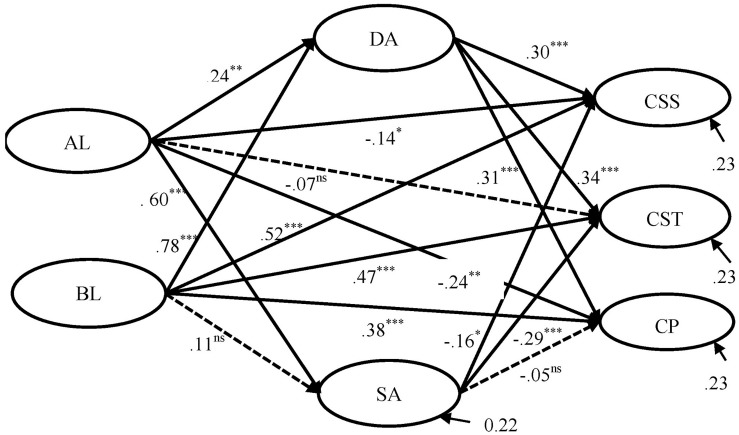
Mediating effect of emotional labor on the effects of paternalistic leadership on teacher commitment. ^∗∗∗^*p* < 0.001, ^∗∗^*p* < 0.01,^ *^p < 0.05, ns, not significant. Dotted lines indicate non-significant paths.

Structural equation modeling was performed to explore the relationship between PL, emotional labor, and teacher commitment. The results are shown in Figure 1. The model reached an excellent data fit (χ^2^ = 1127.84, *df* = 507, RMSEA = 0.051, CFI = 0.97, TLI = 0.97). The results revealed that AL negatively influenced teachers’ commitment to school (β = −0.14, *p* < 0.05) and commitment to the profession (β = −0.24, *p* < 0.01). AL positively predicted both deep acting (β = 0.24, *p* < 0.01) and surface acting (β = 0.60, *p* < 0.01).

Benevolent leadership had a significant effect on deep acting (β = 0.78, *p* < 0.001), commitment to school (β = 0.52, *p* < 0.001), commitment to students (β = 0.47, *p* < 0.001), and commitment to the profession (β = 0.38, *p* < 0.001). Deep acting significantly influenced all three aspects of teacher commitment, while surface acting was a negative predictor for teacher commitment to school (β = −0.16, *p* < 0.05) and commitment to students (β = −0.29, *p* < 0.001).

### Mediation Analysis

The mediating effects of emotional labor strategies were further examined by bootstrap analysis, and the results are shown in Table 2. According to Hayes (2009), the indirect effect is significant if zero is not between the lower and upper bound in the 95% confidence interval. Surface acting negatively mediated the effects of AL on teacher commitment to school (β = −0.10, *p* < 0.001), commitment to students (β = −0.17, *p* < 0.001), and commitment to the profession (β = −0.03, *p* < 0.001). Deep acting significantly mediated the effects of BL on teacher commitment to school (β = 0.23, *p* < 0.001), commitment to students (β = 0.26, *p* < 0.001), and commitment to the profession (β = 0.25, *p* < 0.001). In addition, deep acting also had positive mediating effects on the relationship between AL and teacher commitment to school (β = 0.07, *p* < 0.01), commitment to students (β = 0.08, *p* < 0.01), and commitment to the profession (β = 0.07, *p* < 0.001). These results partly supported hypothesis 3, and surface acting played different mediating roles from deep acting.

**TABLE 2:** Mediation analysis of emotional labor on the effects of paternalistic leadership on teacher commitment.

		Mediation analysis				
					95% CI	
Dependent variable	Independent variable	Mediation variable	Estimates (SE)	*p*	Lower	Upper
CSS	AL	SA	−0.10(0.02)	0.000	[−0.12, −0.07]	
		DA	0.07(0.03)	0.008	[0.02, 0.11]	
	BL	SA	−0.02(0.02)	0.119	[−0.04, 00]	
		DA	0.23(0.03)	0.000	[0.19, 0.28]	

CST	AL	SA	−0.17(0.03)	0.000	[−0.22, −0.13]	
		DA	0.08(0.03)	0.004	[0.04, 0.13]	
	BL	SA	−0.03(0.02)	0.126	[−0.07, 00]	
		DA	0.26(0.05)	0.000	[0.19, 0.35]	

CP	AL	SA	−0.03(0.01)	0.000	[−0.04, −0.02]	
		DA	0.07(0.02)	0.000	[0.04, 0.11]	
	BL	SA	−0.01(0.00)	0.111	[−0.01, 0.00]	
		DA	0.25(0.03)	0.000	[0.19, 0.30]	

*p < 0.05, **p < 0.01, ***p < 0.001.

## Discussion

Increasing evidence has revealed that the practices of emotional labor and leadership vary widely across East and West cultures (Walker and Qian, 2018; Zheng et al., 2018; Yang et al., 2019). This study contributed to the field of school leadership and emotional labor in two aspects. First, the emotional interactions between leaders and followers have been underexplored, especially how leadership styles influenced followers’ emotions (Hülsheger and Schewe, 2011; Berkovich and Eyal, 2015). Second, the current school leadership field was still dominated by leadership theories originating from Anglo-American contexts, and it is necessary to explore more culturally specific leadership styles and mechanisms in various contexts (Hallinger, 2011; Chen et al., 2014; Walker and Qian, 2018; Hiller et al., 2019). This study examined the emotional interactions between school leaders and teachers in a Chinese context, with a particular interest in a culturally specific leadership style, PL. This study expanded our understanding of how authoritarian and BL behaviors can induce different emotional responses from followers through a quantitative method in school contexts.

### PL and Its Consequences

Researchers found a positive relationship between benevolence and subordinate outcomes (Chen et al., 2014; Bedi, 2019). As expected, BL behavior can positively predict a teacher’s commitment to school, commitment to students, and commitment to the profession, which is similar to most studies conducted in other contexts (Farh et al., 2008; Chen et al., 2014; Wang and Guan, 2018). By contrast, the results showed that AL had negative effects on teachers’ commitment to school and commitment to the profession. Furthermore, when comparing the beta weight of two dimensions of PL, the results lend credence to the argument that BL showed greater dominance over AL in predicting follower outcomes, including job satisfaction, commitment, and performance (Bedi, 2019). When teachers characterize their leaders as high benevolence and low authoritarianism, they are more inclined to attach to the school and teaching profession. Although early studies (e.g., Redding, 1990) of PL suggest the necessity of authoritarianism for the effectiveness of subordinates’ performance, along with the influence of rapid economic growth and social transformation, great changes in social culture and people’s traditional concepts have taken place, and the desire for fairness has become the modern common pursuit (Wu et al., 2012). Thus, we agree with Farh et al.’s (2008) observation that in school contexts, most teachers “expect their principals to be high benevolence and low authoritarianism” (Farh et al., 2008, p. 186).

Paternalistic leadership had different effects on teacher emotional labor strategies. Specifically, BL can significantly predict deep acting. Previous studies showed that general leadership practices (e.g., developing people, concern for teachers) could increase teachers’ use of deep acting (Zheng et al., 2018), help followers to cope with negative events, or transform negative moods into improved performance (McColl-Kennedy and Anderson, 2002). The results imply that principals’ caring, concern, encouragement, and understanding of the real cause of teachers’ unsatisfied performance may help teachers to rethink deeply about the situation. In contrast, AL can enhance teachers’ surface acting. Previous studies showed that authoritarian behaviors might cause negative emotions such as fear and anger, which cause subordinates to suppress their emotions (Wu et al., 2002; Farh et al., 2006; Chen et al., 2014). Further, the results showed that AL can also positively influence teachers’ deep acting strategy, which was unexpected and will be further explained in the following section.

### The Role of Emotional Labor

We further examined the role of emotional labor. Previous studies showed that deep acting may be beneficial while surface acting might result in negative outcomes (Hülsheger and Schewe, 2011; Humphrey et al., 2015; Grandey and Melloy, 2017). As expected, deep acting can facilitate teachers’ commitment to school, commitment to students, and commitment to the profession, and surface acting had negative influences on teacher commitment to students and commitment to school. Previous studies showed that deep acting had positive effects on organizational commitment (Hülsheger and Schewe, 2011).

The mediation analysis showed that surface acting played a negative role in AL and teacher commitment to students and commitment to school. When interacting with authoritarian leaders, teachers may be afraid to express true emotions (i.e., fear, anger) and they fake their emotions. Previous studies found that AL may suppress subordinates’ emotional expression (Wu et al., 2002; Farh et al., 2006), and our findings partly support this argument. Authoritarian behaviors may lead teachers to suppress their emotions and then cause negative effects on their attachment and identification with the school and students.

Deep acting positively mediated the effects on BL and teacher commitment, which we expected. The results mean that benevolent behaviors enhance teacher commitment through facilitating teachers to modify their felt emotions. This finding echoed some previous studies that found that deep acting played a significantly mediating role on the effect of leadership practices on teachers’ teaching efficacy (Zheng et al., 2018). Benevolent leaders attach importance to maintain the good relationships with teachers, and principals’ caring, concern, encouragement, and understanding of the real cause of teachers’ unsatisfied performance may help teachers to modify their own inner feelings (Berkovich and Eyal, 2015; Zheng et al., 2018). Thus, principals’ benevolent behaviors may help teachers to better cope with their emotions in work (Gooty et al., 2010), to increase their passion for their job and reduce their fear (Berkovich and Eyal, 2015; Zheng et al., 2018), and to transform bad moods into positive work attitudes (McColl-Kennedy and Anderson, 2002).

It should be noted that the study showed that AL had positive effects on deep acting strategy, and deep acting had positive mediating effects between AL and teacher commitment to school, commitment to students, and commitment to the profession. The findings showed that authoritarian behaviors might enhance teacher commitment through promoting teachers’ deep acting, which revealed the two-sided effects of AL. Although AL had a direct negative influence on teacher commitment to school and commitment to the profession, these effects can be transformed as positive effects through the mediating role of deep acting strategy. Despite a general consensus in the literature that authoritarian tendencies are associated with negative behaviors and outcomes (Pellegrini and Scandura, 2008; Chen et al., 2014; Bedi, 2019), some recent studies acknowledge that for some outcomes and in some situations, authoritarianism may be positive (Tian and Sanchez, 2017; Harms et al., 2018; Wang and Guan, 2018). For example, researchers found that authoritarian leaders offer a better sense of what it means in terms of attitudes, emotional response, and behaviors as a member of the team (Rast et al., 2013; Schaubroeck et al., 2017). Some authoritarian behaviors can help employees gain a better understanding of what they should and should not do within the group (Wang and Guan, 2018). The results mean that principals being strict with teachers, by scolding teachers when they make mistakes or fail to reach expected targets, may help teachers to reflect on their emotions, to think of their emotional experience in school, and to create positive outcomes.

## Limitations and Implications

When interpreting our findings, some limitations should be kept in mind. First, the sample size is relatively small, and there exist striking differences in different regions in a big country like China. Although we used a random sampling strategy to collect data, the results may not be generalized to all schools in China. Thus, future research should expand the sample size by including participants from different subjects, grade levels, and regions. Second, this cross-sectional nature of the study precludes us from making definite casual conclusions. Further studies are suggested to focus on generalizing the results using a longitudinal method. In addition, as both leadership practices and emotional labor are influenced by cultures and contexts, future studies can explore the process of how different PL strategies affect teachers’ emotion work in specific situations. Hence, qualitative methods or a mixed-method design could be used in future studies.

The findings have some implications for principals “leading with teacher emotional labor” (Humphrey et al., 2008; Zheng et al., 2018). First, the influences of PL on teachers vary. BL practices had direct and positive effects, while authoritarian behaviors had negative effects on teacher commitment. We suggest that leaders in school contexts acting as paternalistic should forgo their use of authoritarianism and rely more on benevolence (Farh et al., 2008; Chen et al., 2014), especially in hierarchical societies such as China. Principals are suggested to “act benevolently toward subordinates while upholding high personal moral standards and exercising little authoritarianism. They lead by winning subordinates’ respect and gratitude and rarely resort to positional authority” (Farh et al., 2008, p. 186).

Second, school leaders are suggested to help teachers with a more comprehensive understanding of the emotional demands of teaching, its potential influences, and possible coping strategies (Yin et al., 2019). The findings showed the benefits of BL for teachers’ deep acting strategy, and it also found the two side effects of AL. Principals’ caring and concern for teachers’ welfare may help them to better cope with the negative emotions at work, rethink their (emotional) problems, reappraise the situation, and improve commitment. AL will enhance teachers’ surface acting, suppress their true emotions, and have negative influences on teacher commitment. In some situations, authoritarianism can enhance teacher commitment through promoting a deep acting strategy. Aycan (2006) argued that paternalism can be authoritative, meaning that although the leader exercises control, the underlying reason is to promote the follower’s welfare. In this sense, some aspects of AL that degraded the individual dignity (i.e., belittling subordinate contributions, tight personal control, insisting on absolute obedience) of the teachers may contradict the development tendency of modern times and should be reduced as much as possible. Other facets of AL (i.e., imposing strict work standards, setting high-performance standards) may still be retained in some contexts and some situations (Farh et al., 2006). These implications may inform educators and school leaders in cultures characterized by collectivism and high power distance as paternalism is most likely to occur in these cultures.

## Data Availability Statement

The raw data supporting the conclusions of this article will be made available by the authors, without undue reservation, to any qualified researcher.

## Ethics Statement

The studies involving human participants were reviewed and approved by Ethics Committee of Faculty of Education at Southwest University. The patients/participants provided their written informed consent to participate in this study.

## Author Contributions

XZ designed the research. XS collected the data and YL analyzed the data. XZ and YL wrote the manuscript.

## Conflict of Interest

The authors declare that the research was conducted in the absence of any commercial or financial relationships that could be construed as a potential conflict of interest.
